# Supplementary material for: Genomic hypomethylation in cell-free DNA predicts responses to checkpoint blockade in lung and breast cancer
Source: Sci Rep. 2023 Dec 18;13:22482. doi: 10.1038/s41598-023-49639-4 (PMC10728099; doi:10.1038/s41598-023-49639-4)
Supplement: Supplementary file 1 — Supplementary Figures. [file 41598_2023_49639_MOESM1_ESM.pdf]

Supplementary Figure 1

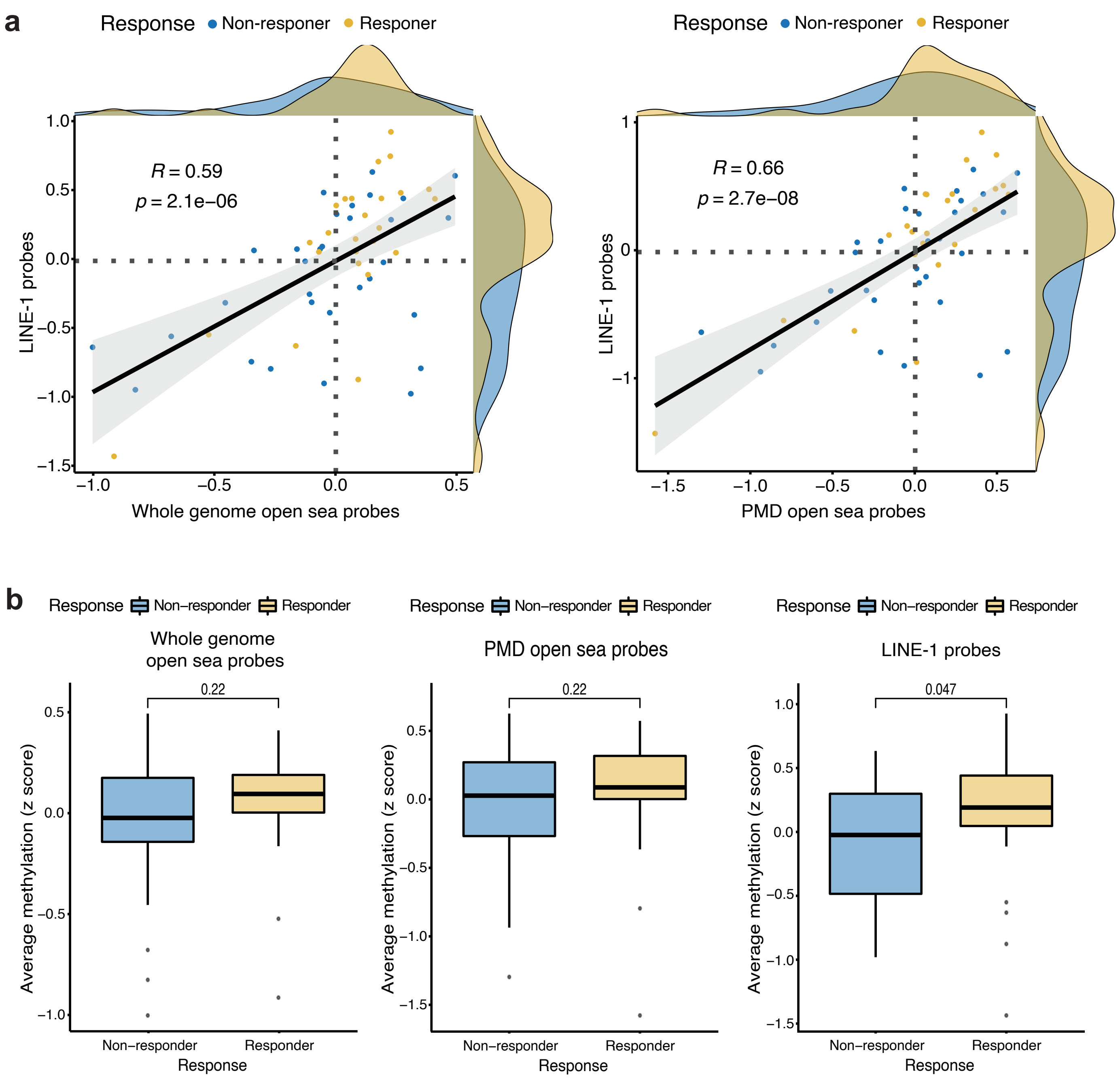

Supplementary Figure 1. Comparison between the estimate of genomic methylation by open sea probes and LINE-1 probes in methylation arrays. (A) Correlation plot between the normalized average methylation values of the whole-genome (left) or PMD (right) open sea probes and those of the LINE-1 probes from lung cancer methylation array data. (B) The power of the whole genome or PMD-based versus LINE-1-based methylation measures from microarrays in distinguishing ICB therapy response in lung cancer.

Supplementary Figure 2

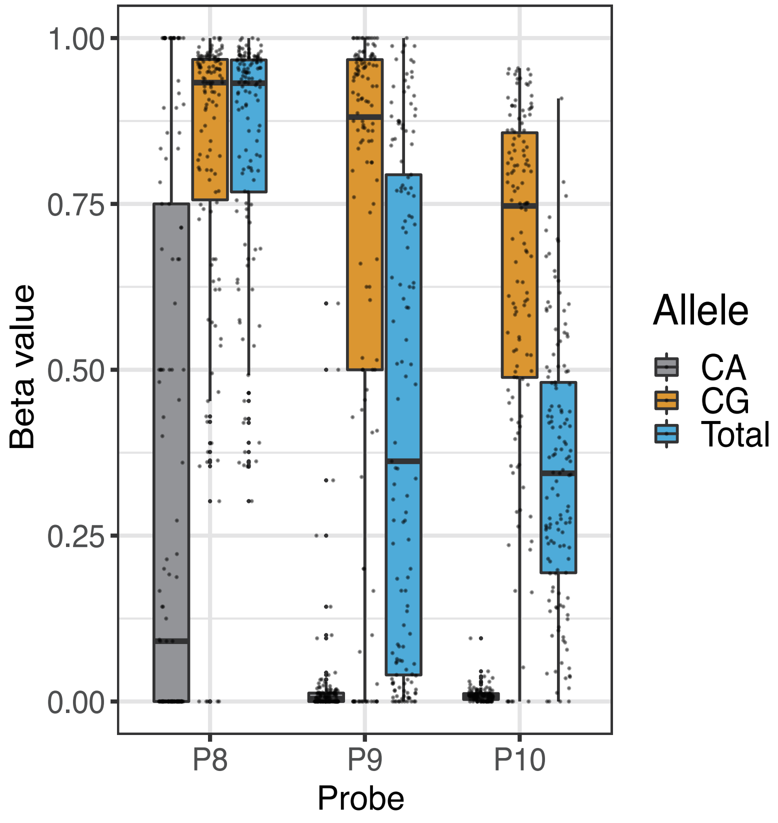

Supplementary Figure 2. Distribution of iMethyl beta values for non-CpG probes. Distribution of cytosine methylation levels at CpA and CpG sites on the three non-CpG array probes measured with iMethyl-tissue in lung cancer.

Supplementary Figure 3

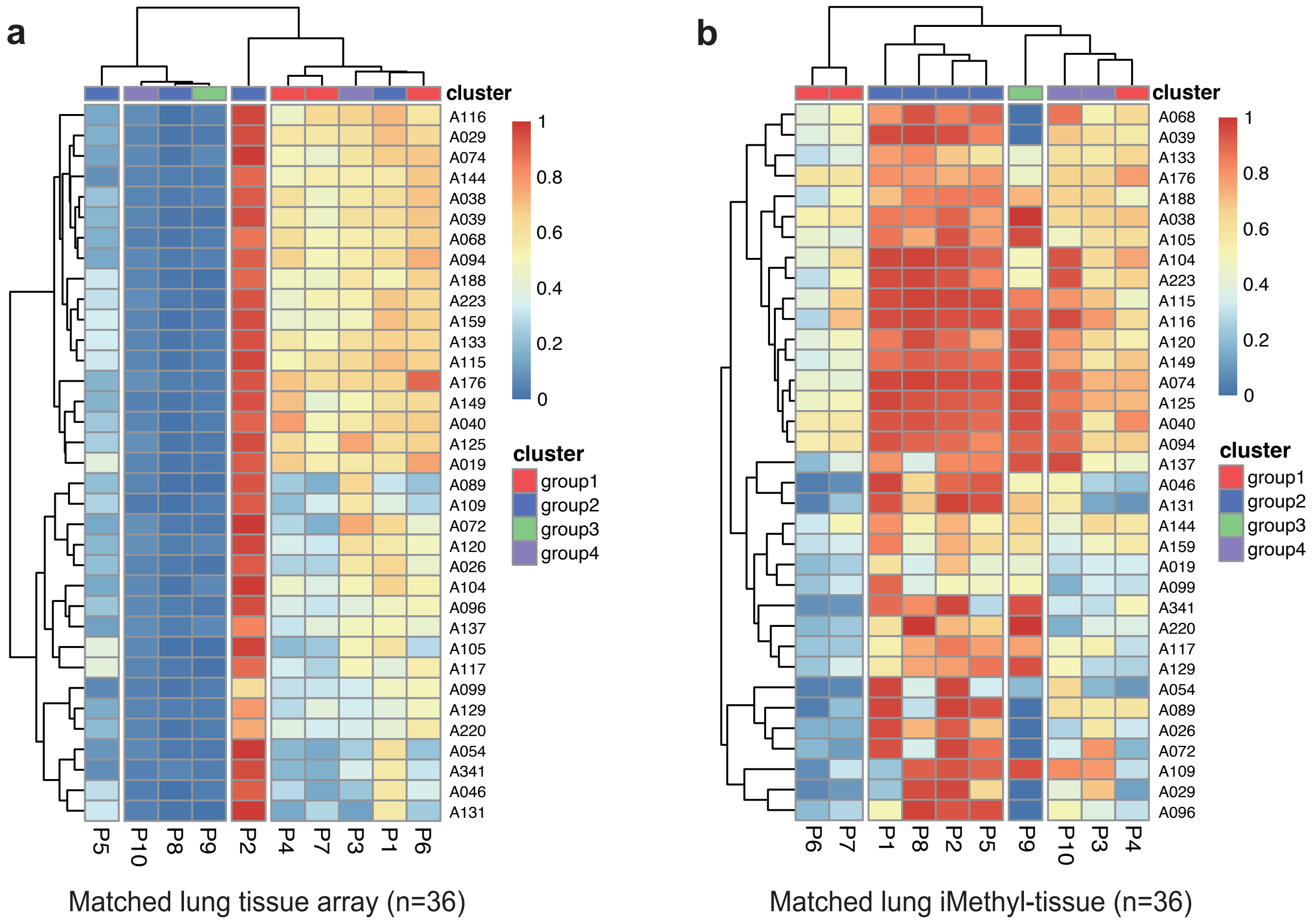

Supplementary Figure 3. Comparison of array-tissue and iMethyl-tissue by hierarchical clustering in lung cancer. Clustering by the methylation patterns based on matched (A) array data and (B) iMethyl data in lung cancer.

Supplementary Figure 4

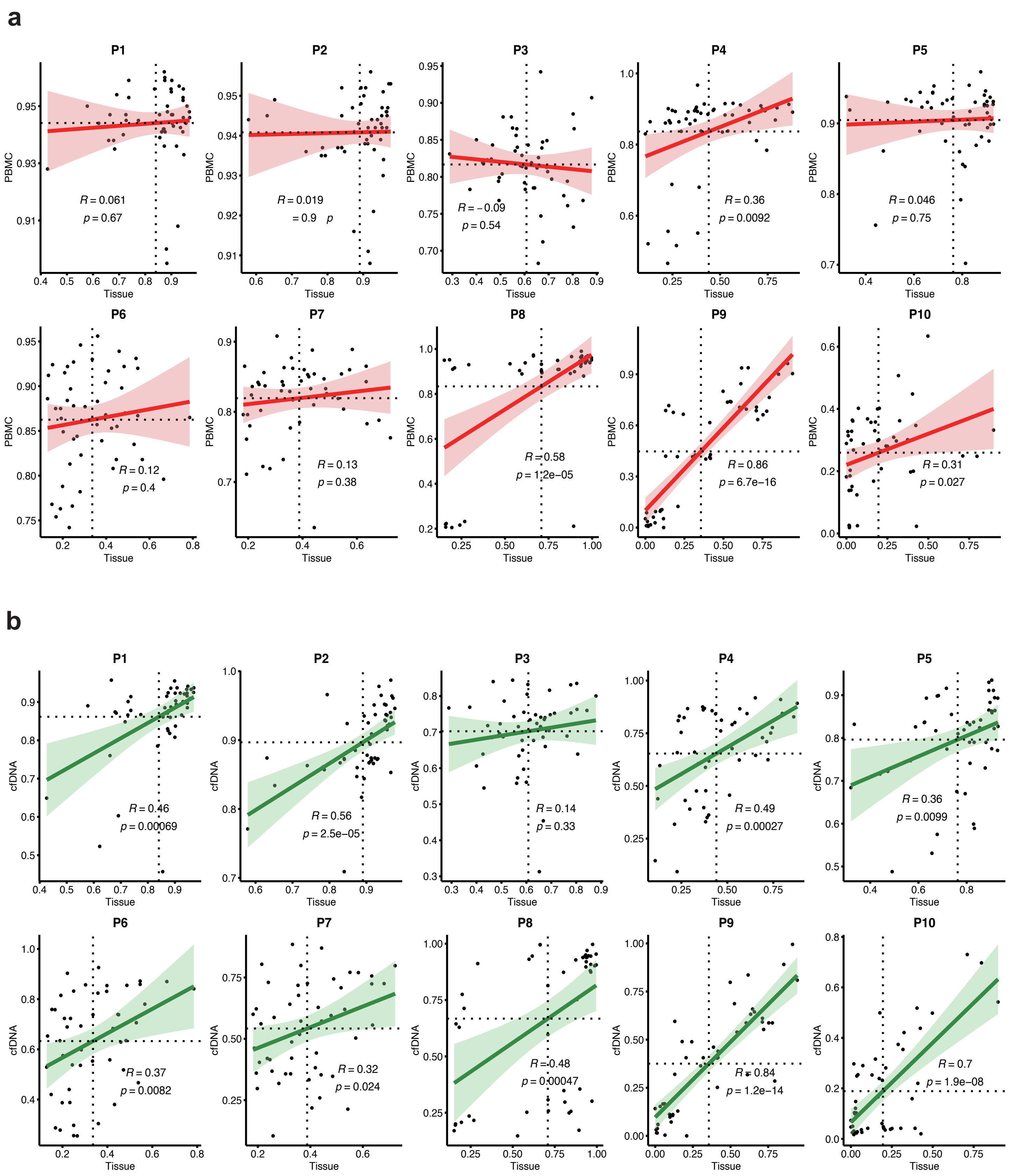

Supplementary Figure 4. Comparison of iMethyl-tissue with iMethyl-PBMC and iMethyl-liquid. (A) Correlation plot per probe between iMethyl-tissue and iMethyl-PBMC. (B) Correlation plot per probe between iMethyl-tissue and iMethyl-liquid.

Supplementary Figure 5

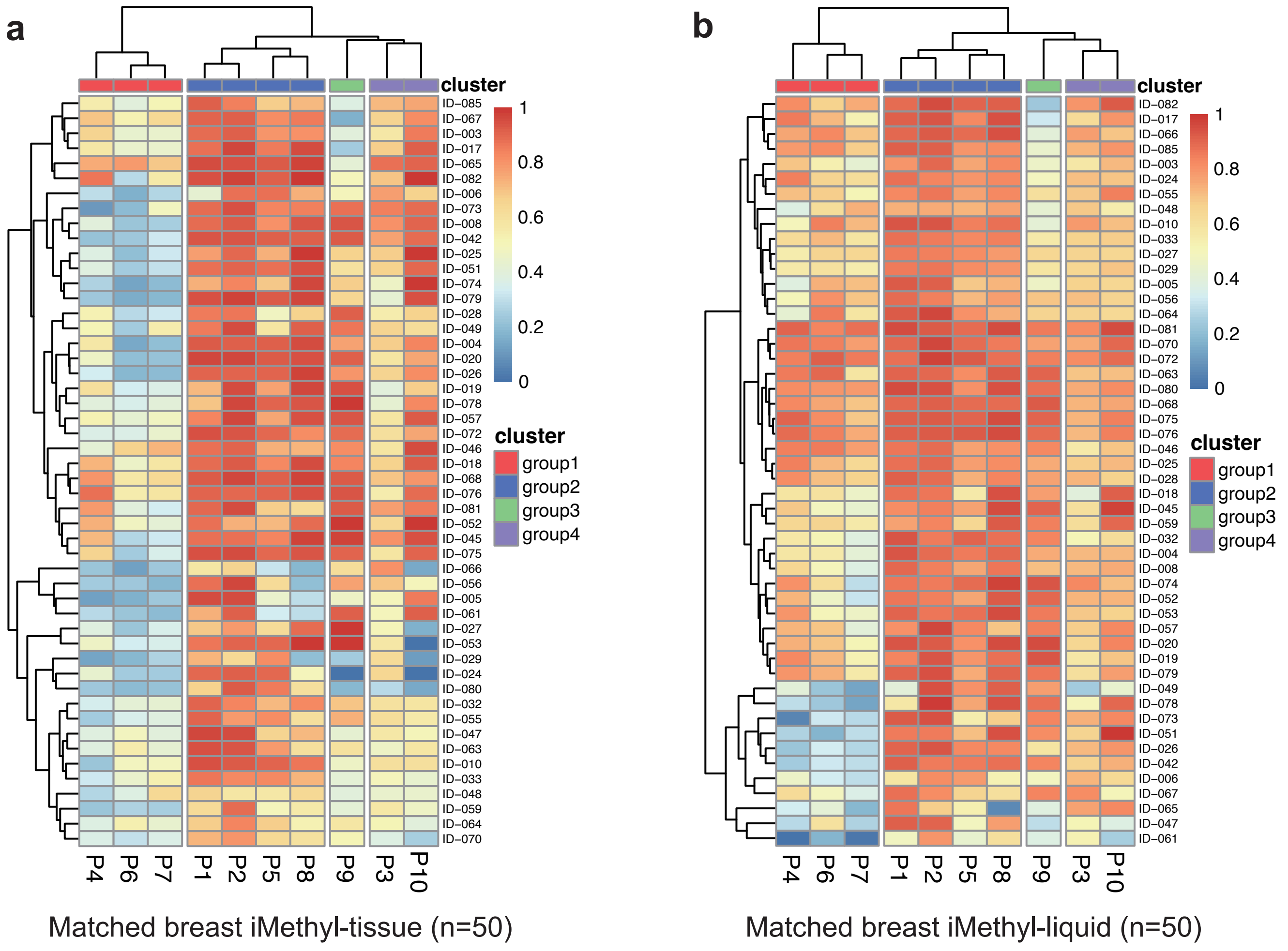

Supplementary Figure 5. Comparison of iMethyl-tissue and iMethyl-liquid by hierarchical clustering in breast cancer. Clustering by the methylation patterns based on matched (A) iMethyl-tissue and (B) iMethyl-liquid in breast cancer.

Supplementary Figure 6

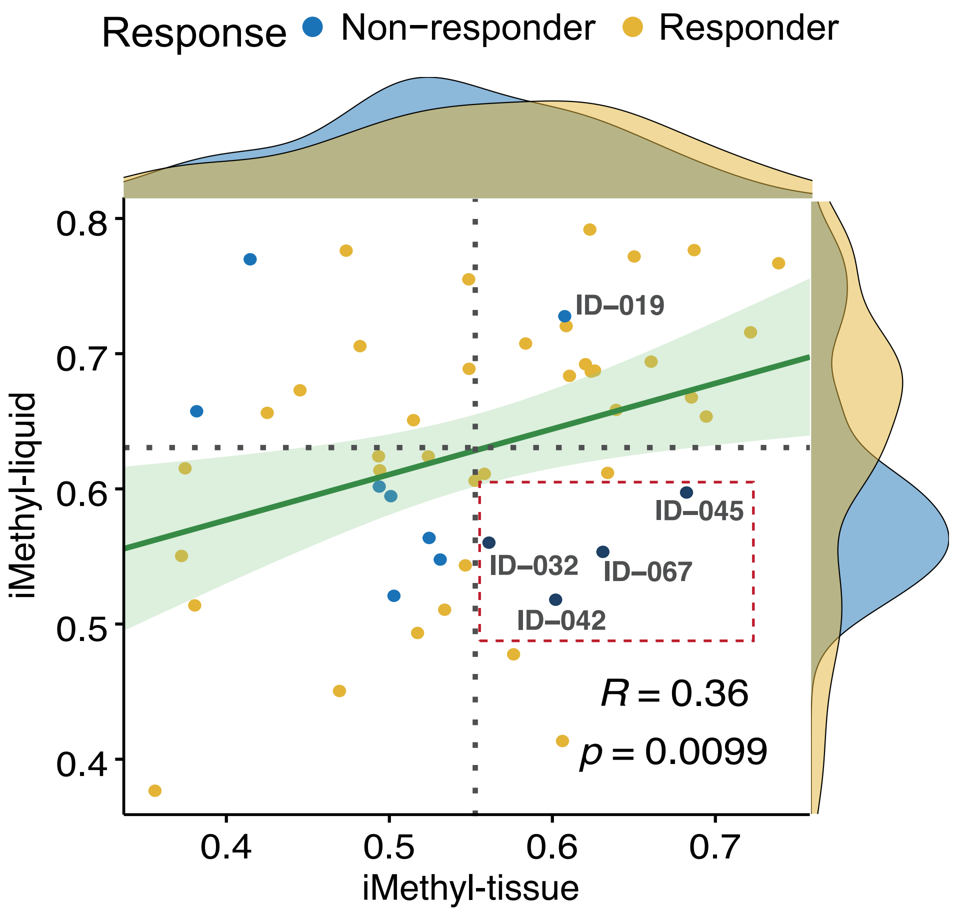

Supplementary Figure 6. Correlation between iMethyl-liquid and iMethyl-tissue for ICB responders and non-responders in breast cancer. Tissue-high and cfDNA-low non-responder samples are enclosed in the red box.

Supplementary Figure 7

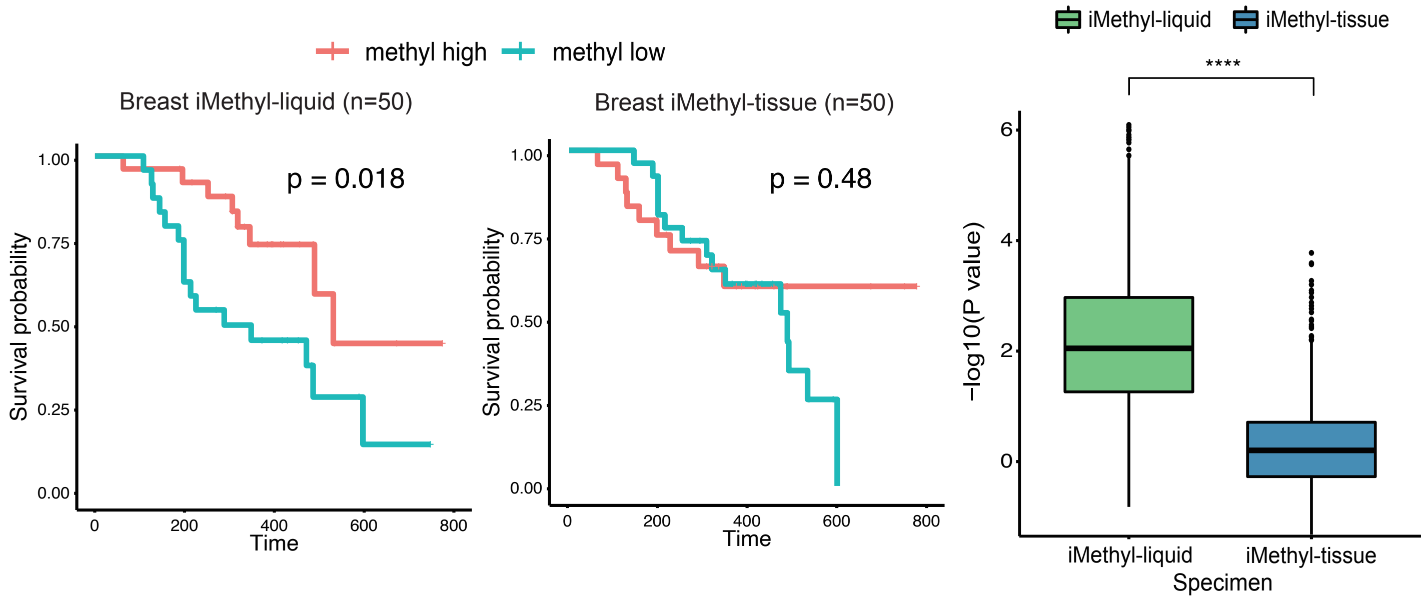

Supplementary Figure 7. Comparison of matched iMethyl-liquid and iMethyl-tissue in predicting ICB efficacy. Survival analysis (left) and bootstrap analysis (right) using the matched breast cancer samples. The survival analysis was performed for the methyl-high and -low group to compare iMethyl-liquid and iMethyl-tissue. For the bootstrap analysis, performance was estimated by 1,000 trials of resampling of individual patient samples. In each sampling, the P value from the survival analysis was obtained. The resulting 1,000 P values for iMethyl-liquid and iMethyl-tissue were compared by the Wilcoxon signed-rank test (P value of **** < 1×10^{-4}).

Supplementary Figure 8

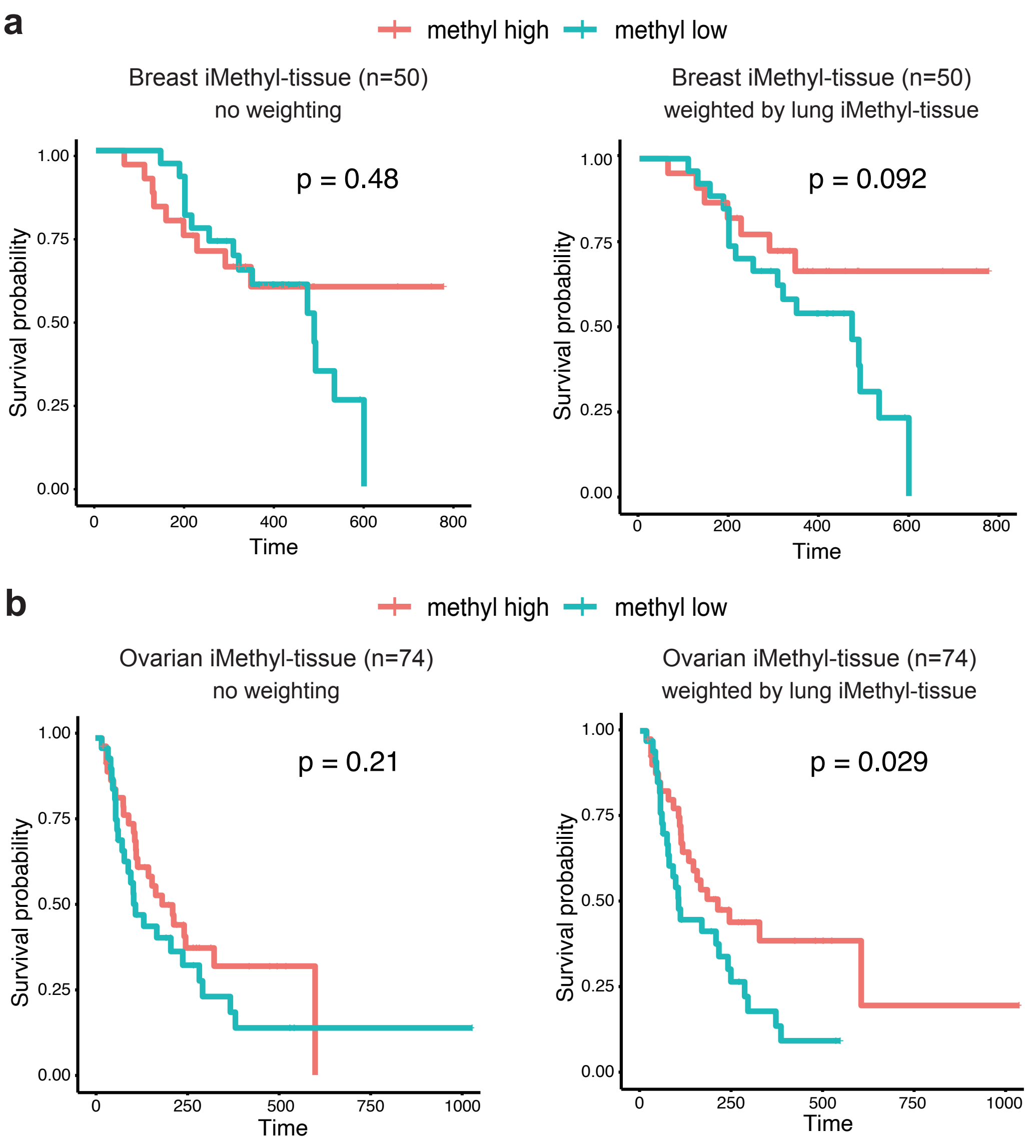

Supplementary Figure 8. Enhanced prediction power of iMethyl-tissue by weighting across cancer types. (A-B) Survival analysis between the methylation-high and -low group based on iMethyl-tissue for our (A) breast cancer and (B) ovarian cancer samples comparing no weighting and weighting by lung cancer iMethyl-tissue.

Supplementary Figure 9

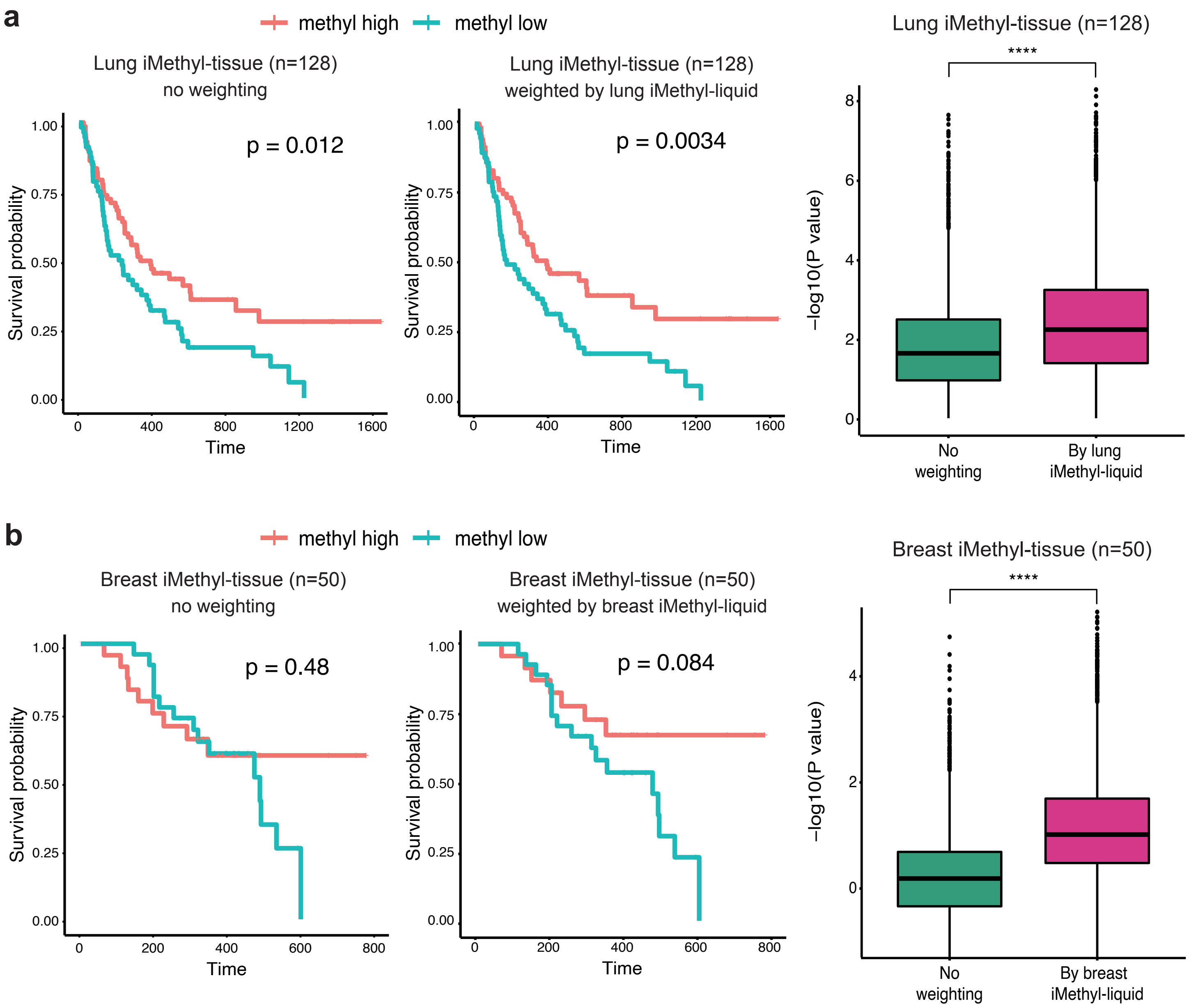

Supplementary Figure 9. Enhanced prediction power of iMethyl-tissue by weighting with iMethyl-liquid. (A-B) Survival analysis (left) and bootstrap analysis (right) for our (A) lung cancer and (B) breast cancer samples. The survival analysis was performed between the methylation-high and -low group based on iMethyl-tissue to compare no weighting and weighting by iMethyl-liquid of the same cancer type. Performance was estimated by 5,000 trials of bootstrapping of individual patient samples. In each sampling, the P value from the survival analysis was obtained. The resulting 5,000 P values for different weighting schemes were compared by the Wilcoxon signed-rank test (P value of **** < $1x10^{-4}$).

Supplementary Figure 10

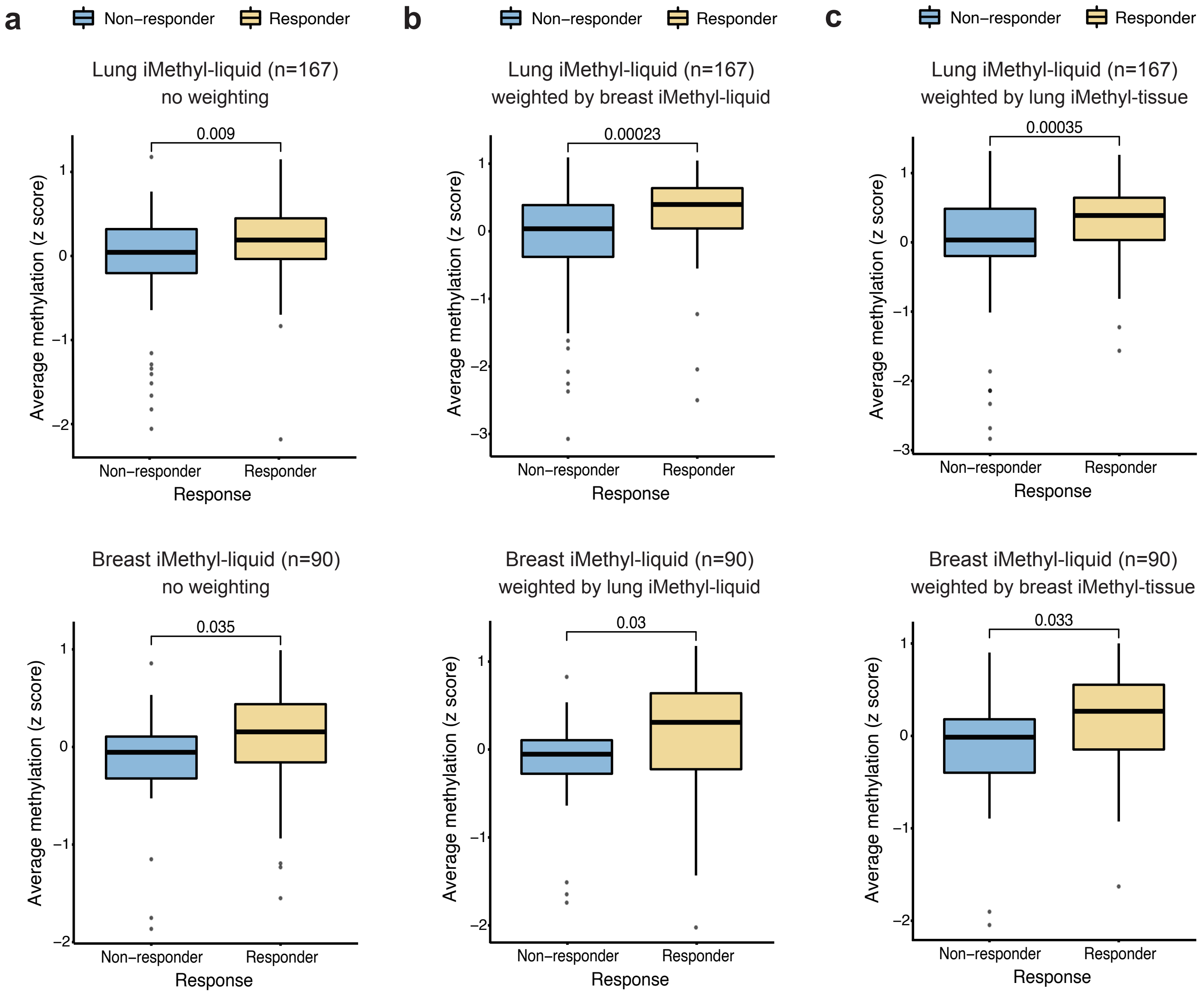

Supplementary Figure 10. Distribution of methylation values between ICB non-responders and responders according to weighting schemes. (A-C) Methylation values according to ICB efficacy from iMethyl-liquid of our lung cancer samples (upper) and breast cancer samples (lower) with (A) no weighting, (B) weighting by iMethyl-liquid of the other cancer type, and (C) weighting by iMethyl-tissue of the same cancer type.

Supplementary Figure 11

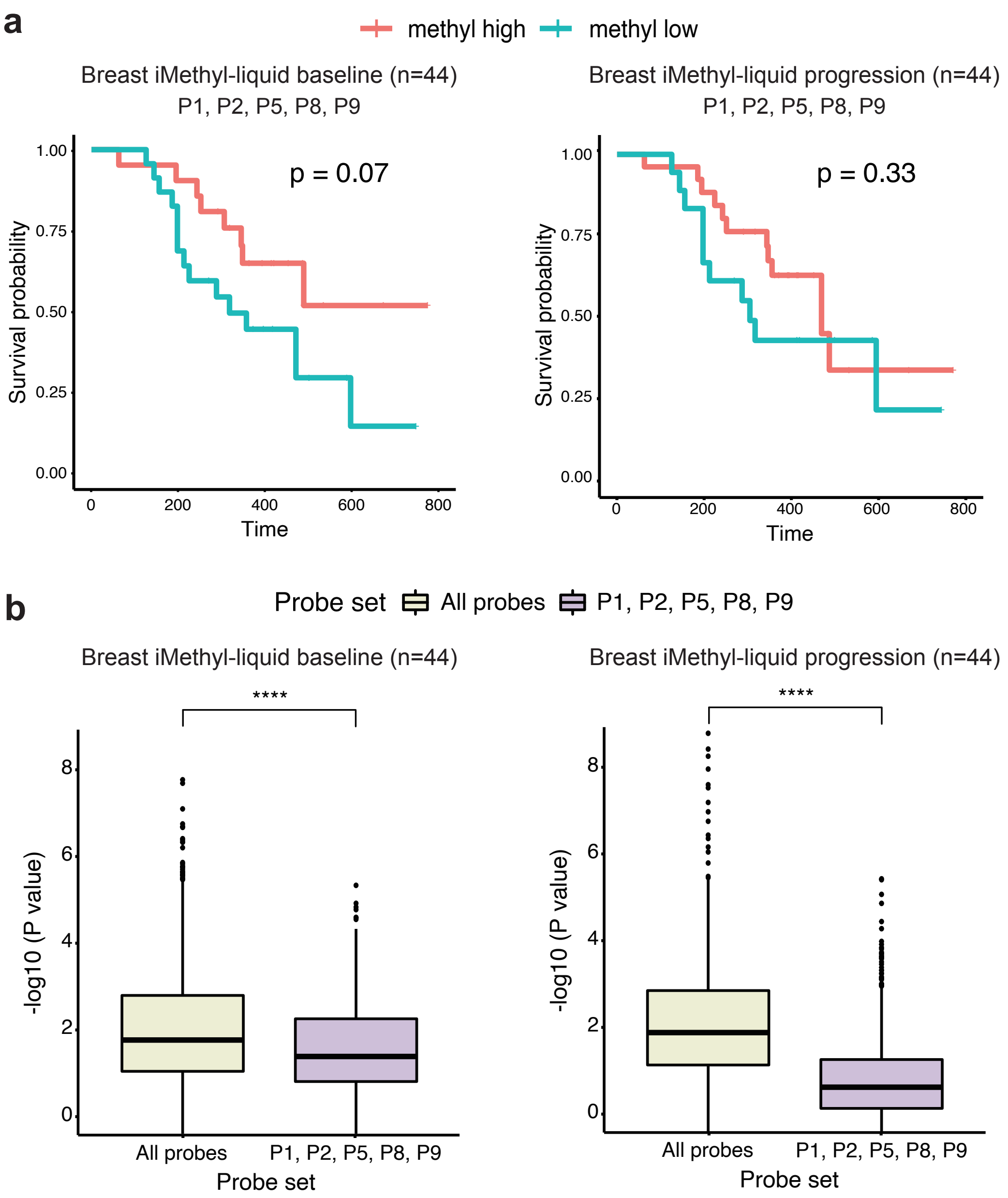

Supplementary Figure 11. Prediction power of iMethyl-liquid using probes with significant changes during disease progression. (A) Survival analysis between the methylation-high and -low group based on the average methylation values of the 5 selected probes at the baseline (left) and progression (right) point. (B) Prediction power measured by bootstrapping analyses comparing the set of the 10 probes and that of the 5 probes at baseline (left) and progression (right). Performance was estimated by 1,000 trials of bootstrapping of individual patient samples. In each sampling, the P value from the survival analysis was obtained. The resulting 1,000 P values for the two probe sets were compared by the Wilcoxon signed-rank test (P value of **** < 1×10^{-4}).

Supplementary Figure 12

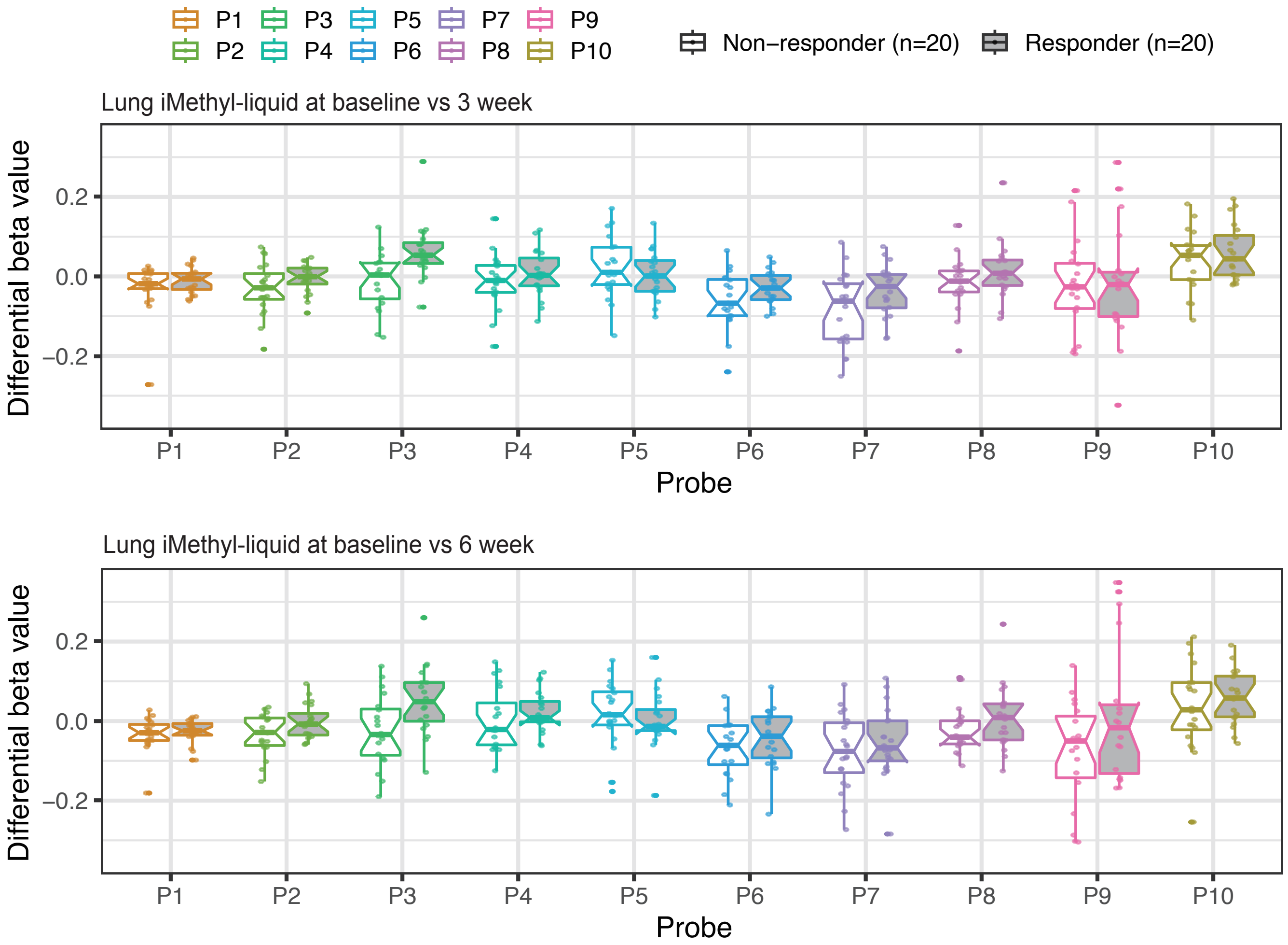

Supplementary Figure 12. EDT methylation changes in association with clinical responses. Differential beta values at each probe between matched baseline and EDT iMethyl-liquid that were compared between the responders (n=20) and non-responders (n=20) of our lung cancer cohort. The EDT sampling was made at 3 weeks (upper) and 6 weeks (lower) after initiating treatment for the same samples.
